# Understanding the Role of CalFresh Participation and Food Insecurity on Academic Outcomes among College Students during the COVID-19 Pandemic

**DOI:** 10.3390/nu15040898

**Published:** 2023-02-10

**Authors:** Brittany M. Loofbourrow, Anna M. Jones, Suzanna M. Martinez, Leslie C. Kemp, Gretchen L. George, Rachel E. Scherr

**Affiliations:** 1 Department of Nutrition, University of California, Davis 1 Shields Ave, Davis, CA 95616, USA; 2 CalFresh Healthy Living, University of California, 1632 DaVinci Court #31, Davis, CA 95618, USA; 3 Department of Epidemiology and Biostatistics, University of California, San Francisco 550 16th St, San Francisco, CA 94158, USA; 4 Aggie Compass Basic Needs Center, University of California, Davis 1 Shields Ave, Davis, CA 95616, USA; 5 Family, Interiors, Nutrition & Apparel, San Francisco State University, 1600 Holloway Avenue, San Francisco, CA 94132, USA; 6 Scherr Nutrition Science Consulting, San Francisco, CA 94115, USA

**Keywords:** food security, higher education, college, Supplemental Nutrition Assistance Program, SNAP, CalFresh

## Abstract

Food insecurity (FI) is associated with many adverse outcomes in college students. The Supplemental Nutrition Assistance Program (SNAP, known as CalFresh in California) has been observed to alleviate FI; however, on college campuses, the benefits of food assistance programs are not well understood. This study investigated whether college students benefit from CalFresh participation. It was hypothesized that students would experience increased FI over time and that CalFresh participation would moderate the effect of FI on grade point average (GPA). A comprehensive FI and CalFresh questionnaires were distributed during the 2020–2021 academic year to 849 students. The chi-square test of independence assessed differences between FI and student factors. A Friedman test assessed differences in FI during the three quarters. Moderation analysis assessed whether CalFresh participation moderated FI’s effect on GPA. Differences were observed among food security scores in Winter 2021 (median = 1.69) and Fall 2020 (median = 2.14; *p* = 0.013) and Spring 2020 (median = 2.17; *p* = 0.009). In the moderation model, the interaction of FI score and CalFresh participation was positively correlated with GPA (B = 0.11; *p* = 0.002). These results indicate that SNAP/CalFresh participation was particularly beneficial for mitigating the negative effects of FI on GPA. Given these benefits, encouraging SNAP/CalFresh enrollment should be a priority for university administrators.

## 1. Introduction

Food insecurity, the lack of access to nutritionally adequate food which supports a healthy and active lifestyle [1], is a growing concern in the college student population [2,3,4,5,6,7,8,9]. Although no group in the United States (US) is immune to food insecurity, college students have been observed to experience food insecurity at a prevalence that can greatly exceed food insecurity in adults nationally, with estimates of four times or more of the 10.5% national prevalence [3,10]. Similar to the general population, food insecurity in college students has been associated with demographic characteristics, including being from a low-income background or belonging to certain racial or ethnic backgrounds [11,12]. Food insecurity has been associated with a suite of negative experiences and conditions, including poor physical health and increased stress; feelings of stigma [13], strained personal relationships [13], and a higher prevalence of anxiety and depression [8,14,15]. Of particular concern in this group is the effect that food insecurity may have on academic outcomes, such as grade point average (GPA) and retention. Compared to their food-secure peers, students experiencing food insecurity have been reported to be more likely to neglect their academics to pursue wage-earning work, drop out of school, and have a lower GPA [16,17,18,19,20]. Following the through line of dampened academic performance, it has been observed that students who achieve at lower levels may go on to experience an altered career trajectory, as employers and universities often screen job or graduate school applicants by college GPA [21,22,23].

Although many factors may contribute to each student’s academic success, current observations of college campuses point to an increased focus on improving basic needs access as a way to support student success [24,25]. At the University of California (UC), the definition of “basic needs” includes “equitable access to nutritious and sufficient food; safe, secure, and adequate housing […]; healthcare […]; affordable transportation; resources for personal hygiene care; and emergency needs for students with dependents [26]”. To improve basic needs access at the UC, all ten campuses have basic needs centers in place which support students experiencing basic needs insecurity [27]. Each UC campus hosts food pantries to provide students with dry goods and fresh produce and nutrition education opportunities [27]. To further supplement these campus efforts, leaders at various UC campuses work closely with their local counties and California State University leadership to provide students with support in determining eligibility and applying for the Supplemental Nutrition Assistance Program (SNAP, referred to as CalFresh in California) [28]. This nutrition assistance program is funded by the US federal government and provides food benefits to eligible low-income individuals across the US. Nationwide, SNAP has been observed to help alleviate food insecurity and financial insecurity [29]. With the double burden of food insecurity and financial insecurity observed on college campuses, food benefits like these may offer a welcome solution to improving basic needs security and improving academic outcomes [25]. Access to this resource in a school setting may be particularly beneficial, as these benefits function year-round, while campus pantries may not be available while campuses are closed [30,31].

On college campuses, the benefits of SNAP are not well-understood due to a lack of research in this area [32]. Given the observed advantages of SNAP benefits in the general population, it is of interest to understand if college students benefit in the same way in terms of improved food security. Further, participation in SNAP may confer additional benefits specific to this population; students with greater food security have been observed to have improved GPAs compared to students experiencing food insecurity [8,16,17], and participation in SNAP may be associated with improvements in GPA [33].

Due to the structure of financial aid disbursements (one disbursement at the beginning of the academic term), it was hypothesized that over the span of the 10-week academic quarter at the study institution, student food security would decrease as financial aid monies were used, which has been observed in a 16-week semester setting [34]. New probes on the data were performed following the rejection of the first hypothesis and observations in food security changes over an academic year. The sub-hypothesis of the study was that CalFresh participation would positively moderate the effect of food insecurity on GPA.

### Study Context

**Figure 1:**
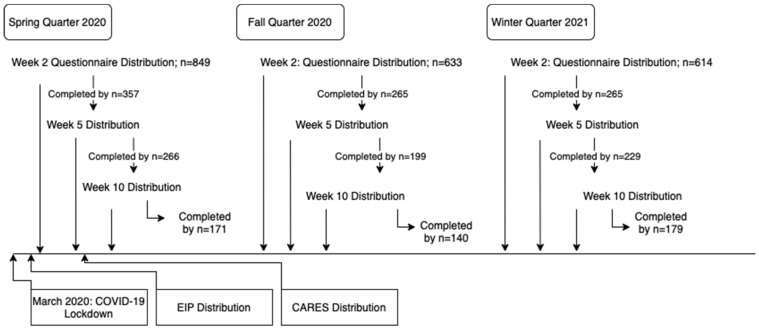
Study Timeline. The EIP Distribution and CARES Distribution refer to the Economic Impact Payment (EIP) and funds from the Coronavirus Aid, Relief, and Economic Security (CARES) Act, respectively.

The current study started at the beginning of the Spring quarter of 2020 (start date 26 March 2020), closely following the World Health Organization’s declaration of the COVID-19 pandemic on 11 March 2020 [35]. Given the unprecedented circumstances of the COVID-19 pandemic and the national increases in food insecurity [36], the initial focus of changes in food security status over time was shifted to examine how CalFresh participation may interact with student food security and affect academic performance, as access to these resources changed with campus closures and on-campus resource operations changing [37,38,39]. In addition to the changes in resources available on campus, COVID-19 relief funds were provided to eligible students during the Spring term of the school year. The Economic Impact Payment and funds from the *Coronavirus Aid, Relief, and Economic Security* (CARES) *Act* were distributed in March and May 2020, respectively, which may have contributed to changes in student food security status [40,41]. Figure 1 details important time points and relevant circumstances.

Given the pervasive issue of college student food insecurity and the pressures introduced by the COVID-19 pandemic, the current study sought to examine how student food insecurity may be moderated by the CalFresh food assistance program.

## 2. Materials and Methods

### 2.1. Sample

The current study was a longitudinal sub-analysis conducted between the months of April 2020 and March 2021. The university’s office of Budget and Institutional Analysis provided the research team with the initial n = 10,000 student sample during the previous academic quarter (January–March 2020) for the initial cross-sectional study, which was representative of the university population based on race/ethnicity, academic class level (including undergraduate and graduate students), college, international student status, and California residency. Out of this population, n = 5000 were representative of the university student body, and the remaining n = 5000 were selected based on the same criteria, and additionally were oversampled for recipients of the federal Pell Grant (provided to students from low-income families earning less than USD 50,000 annually) to ensure that students exhibiting financial need and likely associated food insecurity were surveyed. Of the initial 10,000 students contacted, 1408 students completed the questionnaire during the previous quarter (Winter quarter, 2020). Of these, 935 students opted to be contacted for future studies; of these, 849 had not graduated or otherwise left the university and were included in the contact list for the Spring quarter of 2020. Students who had graduated or left the university were removed from the contact list for the subsequent study periods, resulting in a contact list of 633 during the Fall quarter of 2020 and 615 during the Winter quarter of 2021. During the Spring quarter questionnaire distribution, n = 171 participants completed all three questionnaires (response rate = 20%). During the Fall quarter distribution, n = 140 participants completed all three questionnaires (response rate = 22%). During the Winter quarter distribution, n = 179 participants completed all three questionnaires (response rate = 29%). Participants were allowed to complete the questionnaires during any quarter they chose, such that participants may have participated in one, two, or all three quarters. The final longitudinal sample included n = 58 participants (overall response rate = 6%).

### 2.2. Study Questionnaire and Data Collection

Questions relating to CalFresh participation and other student lifestyle questions were developed and edited with the help of a panel of content and survey design experts [33]. Two rounds of cognitive interviews [42] were conducted with university students (n = 15; n = 10) to determine whether questions were being answered as intended and to improve clarity. The final draft of questions was reviewed again by the same panel of experts. The questionnaire contained 68 items in total, including the 10-item USDA Adult Food Security Survey Module (USDA AFSSM) [43] and questions about CalFresh eligibility and participation.

The study questionnaire was administered at three time points during the Spring 2020, Fall 2020, and Winter 2021 academic quarters (nine time points in total) using a modified Tailored Design Method [44]. At the beginning of the second week of each academic quarter, potential participants received an initial email invitation to participate, which provided detailed study information, informed consent letter, and a personalized questionnaire link. Participants received the same email containing informed consent documentation and the questionnaire at two additional time points, including the fifth week and tenth week of each academic quarter. The questionnaire was distributed via Qualtrics (Provo, UT, USA) software. In each questionnaire, students electronically consented by providing a university-issued student ID number. Participants who completed all three questionnaires by the end of each academic quarter were given a USD 10 gift card incentive at the end of the academic term.

After data collection via Qualtrics was complete, data were returned to the office of Budget and Institutional Analysis to be deidentified and combined with student-specific demographic and academic data, including age, sex, race/ethnicity, transfer student status (students transferring from a 2-year or another 4-year institution), low-income status (students whose university application indicates a household income below 185% of US federal poverty guidelines), international student status, first-generation status (students whose parents did not complete a 4-year degree), cumulative and quarter grade point average (GPA), college and major, and academic class level.

### 2.3. Data Analysis

Descriptive statistics were used to examine demographic and student characteristics. Chi-square analysis of independence was used to determine if there were differences in food security status among demographic groups between the longitudinal sample and the three-quarter samples. A Friedman test was run to determine if there were differences in mean academic term food security scores over the span of three quarters. A moderation analysis using the PROCESS macro was used to assess whether participation in campus food assistance programs or CalFresh moderated the effect of food insecurity in the Spring quarter and the Fall quarter GPAs; food assistance program participation was not measured during the Winter quarter of 2021. Quarter GPA was transformed by inversion to achieve normal distribution, as determined by visual analysis of Q–Q plot. All statistical analyses were performed using IBM SPSS version 27 (Armonk, NY, USA).

### 2.4. Variables

Independent variables included CalFresh participation and food security status. Participants indicated whether they were currently participating in CalFresh (“yes”/“no”/“not sure”). Participants who indicated “not sure” were coded as “no.” Food security status as measured by 10-item USDA AFSSM was self-reported by participants in the last 30 days. Food security status was defined as follows: high food security (raw score zero), marginal food security (raw score 1–2), low food security (raw score 3–5), and very low food security (raw score 6–10). Low food security and very low food security were collectively referred to as “food insecure”. Quarter GPA based on institutional records was the dependent variable of the study. The following covariates were included: race/ethnicity, age, first-generation student status, transfer student status, low-income status, international citizenship, out-of-state residency, and academic class level (including freshman (0–44.99 units accumulated), sophomore (45–89.99 units), junior (90–134.99 units), senior (135+ units) students and graduate/professional students).

### 2.5. Ethical Standards Disclosure

This study was conducted according to the guidelines in the Declaration of Helsinki and all procedures involving research study participants were approved by the University of California, Davis Institutional Review Board.

## 3. Results

### 3.1. Sample

The Spring quarter sample included n = 171 participants, the Fall quarter sample included n = 140 participants, and the Winter quarter distribution included n = 179 participants. These quarterly samples included only participants who completed all three questionnaires during the quarter.

The longitudinal sample included 58 participants that completed the questionnaires at each of the nine time points (three questionnaire distributions per academic quarter). In this longitudinal sample, 69% of participants were identified as female, 29% as East Asian, 28% as white/Caucasian, 16% as Latino(a)/Chicano(a)/Hispanic, 10% as Middle Eastern/South Asian, with other ethnicities comprising the remaining 17% of participants (Table 1). Nearly half of the participants identified themselves as first-generation students (46%). Institutional records identified 12% of participants as transfer students, 45% as low-income students, 3% as international and out-of-state residents, and 85% as undergraduate students during the Fall 2019 student census. During the Spring 2020 quarter, there were significant differences in the number of out-of-state students, freshmen, and senior students compared to the longitudinal sample. No differences between the Fall 2020 and longitudinal sample were observed; differences in students identified as Native Hawaiian/Pacific Islander and low-income were observed in the Winter 2021 quarter compared to the longitudinal sample.

### 3.2. Changes in Food Security

**Figure 2:**
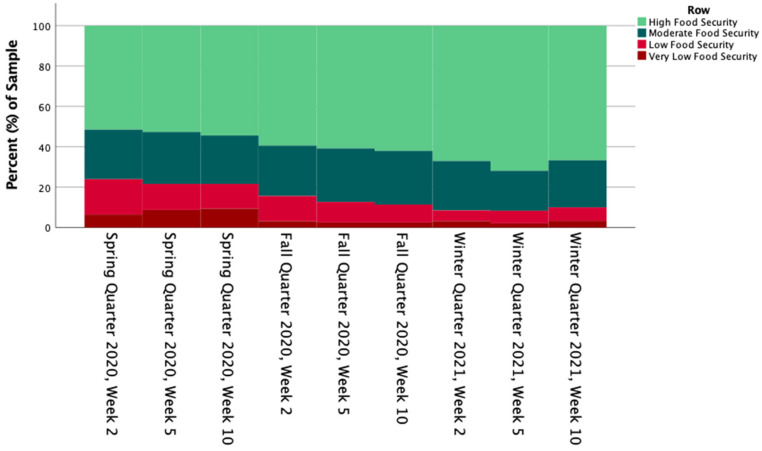
Food Security Status at Each Measurement Timepoint.

With respect to the primary hypothesis of investigating changes in food security over the span of an academic quarter, after the overall Friedman test significance of the longitudinal sample confirmed differences in food security over the span of the academic year (n = 58; χ^2^(2) = 17.008; *p* < 0.001), pairwise comparisons were performed with a Bonferroni correction for multiple comparisons (Figure 2). Differences were observed between average food security scores in Winter 2021 (median = 1.69) and Fall 2020 (median = 2.14; *p* = 0.013; adj. *p* = 0.039) and Winter 2021 and Spring 2020 (median = 2.17; *p* = 0.009; adj. *p* = 0.026). There was no significant difference observed between Spring 2020 and Fall 2020 average food security scores.

Post-hoc Friedman analyses indicated differences in raw quarter food security scores. The overall test significance (χ^2^(2) = 22.607; *p* = 0.004) and pairwise comparisons were performed with a Bonferroni correction. The food security score was different between the Winter 2021, week 5 measurement (median = 4.31) and the Spring 2020 week 2, measurement (median = 5.47) (*p* = 0.020, adj. *p* = 0.733). There was no significant difference observed between all other food security score measurements.

### 3.3. CalFresh Participation Moderation of Food Insecurity

In a regression-based moderation model, the average food security score was significantly correlated with a decrease in quarter GPA specifically for the Spring and Fall 2020 quarters (Appendix A and Appendix B). During the Spring 2020 quarter, CalFresh participation was not correlated with GPA; however, the interaction of average food security score and CalFresh participation was observed to be positively correlated with quarter GPA, indicating a positive moderating effect. These observations remained true after transforming the quarter GPA variable to normalize the outcome variable’s distribution, and in addition, CalFresh participation was negatively correlated with GPA during Fall 2020.

**Figure 3:**
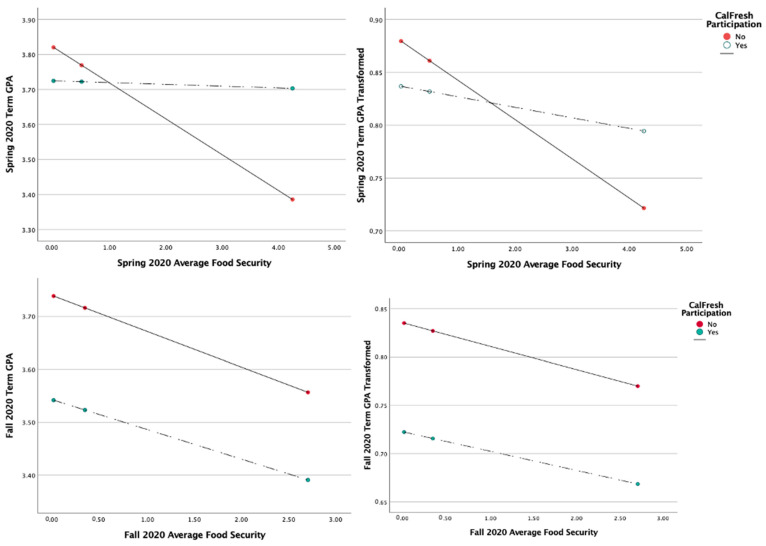
Full Moderation of CalFresh Participation on Average Food Security Score effect on Quarter GPA.

When including demographic and academic covariates into the moderation models, the average food security score remained negatively correlated with quarter GPA during the Spring and Fall of 2020 (Table 2; Figure 3). As in the simple moderation model, the interaction of average food security score and CalFresh participation was observed to be significant, indicating a positive moderating effect of CalFresh participation on food security’s effect on GPA.

After transforming the quarter GPA, the moderation effect of CalFresh participation was no longer significant during Spring 2020 and increasing academic class level had a positive correlation with the quarter GPA. During the Fall 2020 quarter, the female sex was correlated with a decrease in GPA.

## 4. Discussion

The purpose of the current study was to assess how food security changes over the span of a 10-week academic quarter in a longitudinal sample. Overall, the results indicated that food security does not change over the span of a 10-week quarter; however, food security was observed to improve over the span of a full academic year. Due to the observed trend of improvements to food security, the authors further probed the data to investigate whether CalFresh participation was a benefit to students and moderated the effect of food security on GPA. These results indicated that during the first academic quarter (Spring 2020) CalFresh participation was a positive moderator of food insecurity, such that students who participated in CalFresh did not see a decline in GPA with increasing food insecurity as did students who did not participate in CalFresh. In the full moderation model, CalFresh was observed to be a positive moderator when accounting for demographic and academic factors, indicating that participating in food assistance programs was beneficial for students irrespective of demographic characteristics, which have been associated with food insecurity and poor academic outcomes [9,11,19,20]. The moderation was not seen during the subsequent Fall quarter, suggesting that the early responses to the COVID-19 pandemic—including campus closure and emergency benefits—may have influenced GPA in this population.

Food security was observed to improve over time from Spring 2020 to the following Winter quarter, and CalFresh was observed to be uniquely supportive of student outcomes during the Spring quarter. In the broader context of this time, as part of the early pandemic response in the Spring of 2020, low-income students and many students who were financially independent of their parents/guardians (who were more likely to be eligible to participate in CalFresh) received COVID-19 relief payments [41]. The federal Economic Impact Payments and funds from the *Coronavirus Aid, Relief, and Economic Security* (CARES) *Act* were both distributed to eligible students during the Spring quarter of 2020, late in March and early in May, respectively [40,41]. In total, eligible undergraduate and graduate students may have received up to USD 2200 during the Spring quarter in addition to any other financial aid or other benefits. Taken together, this additional financial support and the observed moderating effect of CalFresh participation on food insecurity point to ways in which the Spring quarter of 2020 was unique in terms of available support for student finances and food security. A significant and striking moderating effect on food security was seen in the Spring quarter when pandemic-related aid was disbursed, while the same effect was not seen the following quarter when pandemic-related payments were not available. It is posited that the observed positive moderation of CalFresh in the Spring quarter is visible because CalFresh benefits were able to be used as supplemental food dollars which promote more healthful eating, rather than the sole source of a student’s food dollars with amounts insufficient to wholly support a healthful eating pattern.

On-campus resources are increasing across campuses in recognition of the issue of student food insecurity, and although they may be useful to students and help bridge the food access gap under normal circumstances, they may fall short when campuses are closed for academic breaks or other unforeseen circumstances [31]. The COVID-19 pandemic forced many university administrators to quickly adapt to an unprecedented and unpredictable situation, and at the University of California, all campuses were closed to students [45]. Those who may have been using the resources provided by basic needs centers, which provided fresh foods, pantry staples, and toiletries, were unable to use the resources in the same capacity [28,38]. The closure of the study institution may also have contributed to some of the observations of the current study, wherein food insecurity was worse during the Spring quarter than Winter, owing possibly to the lack of access to campus food supports.

Although the basic needs centers worked to adapt to the changes and continue providing resources to students, they could only serve those students who remained local and chose to participate in the resources [38]. Prior to the institutional closure, the study population had access to multiple basic needs and food resources, including housing case management services, emergency financial aid, campus-wide and graduate student pantries, twice-weekly fresh produce distribution, and satellite pantries serving specific groups (including student families and nonresidents) [28]. Students who left campus (an estimated half of the university’s undergraduate and nearly a third of its graduate population) [46] to return home or otherwise, chose not to use the adapted resources may have experienced a shift in their food security [37,39]. For some students, this shift may have been beneficial—as the academic year progressed, student food security was observed to improve, suggesting that for at least some participants, leaving the university setting improved their food access. However, food insecurity did not disappear entirely from campus, and the sudden loss of campus-based resources during the pandemic closures highlighted areas where on-campus resources may fall short under normal circumstances; while beneficial during the academic year, the lack of resources during term breaks may negatively impact some students’ food security [30].

CalFresh—and SNAP, nationally—may be uniquely useful to vulnerable college students in this area. Although studies of student food security are growing in number and scope, few have examined the intersection of program participation, food security, and academic performance in this population [32,33]. The results of the current study indicate that CalFresh was particularly beneficial during atypical and uncertain academic times. During Spring 2020, the study population was unable to be present on campus, and resources were unavailable, CalFresh participation was observed to moderate the effect of decreasing food security on GPA, such that students who participated in the program saw very little decline in their term GPA compared to those who did not participate.

Still, it is important to recognize that although CalFresh was beneficial early in the pandemic response when pandemic-related funds were available, the same benefit was not observed in the subsequent quarter, suggesting that CalFresh benefits alone may not be enough to provide complete basic needs security and put students experiencing food insecurity on equal footing with their food secure peers. Previous research has described that the cost of higher education has outpaced earnings and grant monies intended to support students, such that the federal Pell Grant is no longer sufficient to cover the cost of higher education [32]. In a practical sense, the cost of attending college is more than federal and state grants can support. To provide for the cost of living (including basic needs like housing and food), students may need to rely on loans in addition to these grants, and these pooled resources may still not be enough to fully support students through their college career [32]. The increasingly precarious financial situation surrounding the modern college experience, coupled with the infusion of COVID-relief funds during the early months of the pandemic, make this Spring quarter an interesting case study for observing how student outcomes may change when they have increased financial stability.

The benefits of improving food security are manifold (improved physical and mental health, and in students, improved academic performance) [9,10], indicating a clear need for food security promotion. CalFresh has the ability to improve food security for millions of individuals, including the growing numbers of college students at risk of experiencing food insecurity, such as low-income, first-generation, and transfer students [2]. Advocacy at the local-, state- and federal- level around college student eligibility and clear access to enrollment is one area where universities can act to improve program participation and impact student food security [47]. By providing resources to build knowledge about these programs on campuses and working alongside SNAP-implementing agencies, universities can positively impact their student populations; by providing students with the resources to improve their food security, they may help students to improve their academic outcomes.

There are several limitations of the current study, including a small sample size, non-normal distributions of GPA and food security scores, and questionnaire distribution timing. Although each quarter saw participants numbering 140–179 participants, only 58 students completed data collection at all time points. Though the moderation models’ outcome variables were transformed to achieve a relatively normal distribution, both quarter GPA and the independent variable of the food security score had non-normal distributions, owing to a ceiling and a floor in the scores, respectively. Results from the current study may not be generalizable to other college students as the study university is on a quarterly academic schedule, which runs ten weeks, compared to 16-week semester schedules. Most importantly, the present study must be considered in the context of the COVID-19 pandemic. Though the authors postulate that CalFresh is a valuable resource for students to improve food security and related outcomes, the results of this study may not represent the student experience under typical academic conditions. Changes in campus food resources and government initiatives, including the EIP grants and funds from the *CARES Act* during this period, may have influenced some of the observed outcomes. Despite these limitations, however, the results of the study contribute to the increasingly complex picture of student food security and the resources in place intended to mitigate its effects.

## 5. Conclusions

The results of this study indicate differences in food security over the span of an academic year, with improvements occurring between Spring 2020 and Winter 2021. Importantly, these findings indicate that early in the COVID-19 pandemic, CalFresh was observed to be a positive moderator for food insecurity’s effect on GPA, with CalFresh participants having a protected GPA despite increased food insecurity. These results indicate the utility of CalFresh for college students, especially in instances where food supports may be limited. Given the observed improvement in academic outcomes, encouraging SNAP enrollment should be a priority for university administrators.

## Figures and Tables

**Table 1:** Food Security, CalFresh Participation, and Demographic Characteristics of Quarter Samples and Longitudinal Sample.

	Spring 2020	Fall 2020	Winter 2021	Longitudinal Sample
	n (%)	n (%)	n (%)	n (%)
Total Sample	171	140	179	58
Food Insecure ^†§^	41 (24)	26 (18.6)	33 (18.4)	-
CalFresh Participation ^†§^	41 (24.0)	28 (20.0)	-	-
Median Quarter GPA	3.88	3.76	3.9	-
Demographic Characteristics				
Median Age	21.0	21.0	21.0	-
Female	125 (73.1)	105 (75.0)	129 (72.1)	40 (69.0)
Race/Ethnicity				
American Indian/Alaska Native	3 (1.8)	2 (1.4)	3 (1.7)	2 (3.4)
Black/African American	3 (1.8)	2 (1.4)	4 (2.2)	1 (1.7)
East Asian	43 (25.1)	42 (30.0)	48 (26.8)	17 (29.3)
Latino(a)/Chicano(a)/Hispanic	37 (21.6)	32 (22.9)	34 (19.0)	9 (15.5)
Middle Eastern/South Asian	10 (5.8)	9 (6.4)	11 (6.1)	6 (10.3)
Native Hawaiian/Pacific Islander	3 (1.8)	2 (1.4)	2 (1.1) *	2 (3.4)
Other Asian	5 (2.9)	3 (2.1)	5 (2.8)	1 (1.7)
Southeast Asian	8 (4.7) ^ab^	11 (7.9) ^a^	19 (10.6) ^ab^	3 (5.2)
White/Caucasian	57 (33.3) ^b^	34 (24.3) ^a^	49 (27.4) ^a^	16 (27.6)
First-Generation Student ^§^	86 (50.3)	67 (47.9)	75 (41.9)	27 (45.8)
Transfer Student	27 (15.8)	17 (12.1)	20 (11.2)	7 (12.1)
Low-Income	65 (38.0)	53 (37.9)	62 (34.6) *	26 (44.8)
International	11 (6.4)	9 (6.4)	9 (5.0)	2 (3.4)
Out-of-State Resident	16 (9.4) *	10 (7.1)	14 (7.8)	2 (3.4)
Class Level (Fall 2019)				
Undergraduate Student	135 (80.1) ^a^	120 (87.1) ^b^	140 (79.3) ^a^	49 (84.5)
Freshman	22 (12.9) *	24 (17.1)	34 (19.0)	13 (22.4)
Sophomore	27 (15.8)	26 (18.6)	35 (19.6)	9 (15.5)
Junior	51 (29.8) ^a^	54 (38.6) ^b^	54 (30.2) ^a^	20 (34.5)
Senior	37 (21.6) ^b^*	16 (11.4) ^a^	17 (9.5) ^a^	7 (12.1)
Graduate or Professional Student	33 (19.3) ^a^	18 (12.9) ^b^	37 (20.7) ^a^	9 (15.5)

Values with different alphabetical superscripts denote the significance of *p* < 0.05. ^§^ Self-reported. ^†^ Measurement from the first time point of each quarter. * Different from the Longitudinal Sample. As these results include individuals from a large sample of unknown students and are a deidentified subset of this list, the identities of individuals belonging to demographic groups with small numbers cannot be identified.

**Table 2:** Full Moderation Model: Food Security Scores’ Effect on Quarter GPA with CalFresh Moderation.

Spring 2020				Spring 2020—Transformed GPA			
	Coeff	SE	*p*		Coeff	SE	*p*
**Average Food Security Score**	**−0.1021**	**0.0223**	**<0.001**	**Average Food Security Score**	**−0.0371**	**0.01**	**0.0003**
CalFresh Participation	−0.0957	0.1135	0.4006	CalFresh Participation	−0.0428	0.0511	0.4041
**Avg Food Security × CalFresh Participation**	**0.0971**	**0.0365**	**0.0088**	Avg Food Security × CalFresh Participation	0.0272	0.0164	0.1008
Ethnicity	0.0136	0.0136	0.3205	Ethnicity	0.0044	0.006	0.4724
Female	−0.0547	0.0809	0.5008	Female	−0.0286	0.0364	0.434
Age	−0.0027	0.0229	0.9049	Age	0.0033	0.0103	0.7528
Transfer Student	−0.0334	0.0963	0.7291	Transfer Student	−0.0429	0.0434	0.3248
First Generation Student	−0.0398	0.0797	0.6187	First Generation Student	−0.0203	0.0359	0.572
Low Income	−0.0215	0.0819	0.7935	Low Income	0.0011	0.0369	0.9762
US Citizen	0.0284	0.1985	0.8863	US Citizen	0.0016	0.0894	0.9861
**CA Resident**	**0.3200**	**0.1582**	**0.0453**	CA Resident	0.0992	0.0712	0.1665
Academic Class Level	0.0857	0.044	0.0538	**Academic Class Level**	**0.0399**	**0.0198**	**0.0465**
Fall 2020				Fall 2020—Transformed GPA			
	Coeff	SE	*p*		Coeff	SE	*p*
**Average Food Security**	**−0.0672**	**0.0239**	**0.0059**	**Average Food Security**	**−0.0241**	**0.0104**	**0.0229**
CalFresh Participation	−0.1966	0.1378	0.1564	CalFresh Participation	−0.1128	0.06	0.0629
Avg Food Security × CalFresh Participation	0.0114	0.0464	0.8062	Avg Food Security × CalFresh Participation	0.0042	0.0202	0.8374
Ethnicity	−0.0197	0.0164	0.2312	Ethnicity	−0.0061	0.0071	0.3914
Female	−0.1187	0.0981	0.229	**Female**	**−0.0898**	**0.0427**	**0.0381**
Age	0.0118	0.0206	0.5683	Age	0.0052	0.009	0.5599
Transfer Student	−0.0413	0.1343	0.759	Transfer Student	−0.0410	0.0585	0.485
First Generation Student	−0.1481	0.0963	0.1272	First Generation Student	−0.0478	0.0419	0.2566
Low Income	0.0401	0.0977	0.6821	Low Income	0.0023	0.0426	0.9576
US Citizen	0.0669	0.2667	0.8024	US Citizen	0.0419	0.1162	0.7191
CA Resident	−0.0513	0.2821	0.8561	CA Resident	−0.0103	0.1229	0.9333
Academic Class Level	−0.0133	0.0475	0.7795	Academic Class Level	0.0103	0.0207	0.6207

Bolded text indicates results with a significance of *p* < 0.05.

## Data Availability

Data sharing is not applicable.

## Appendix A

Simple Moderation of CalFresh Participation on Average Food Security Score on Quarter GPA.

## Appendix B

Simple Moderation Model: Food Security Scores’ Effect on Quarter GPA with CalFresh Moderation.

**Table:**

Spring 2020				Spring 2020—Transformed GPA			
	Coeff	SE	*p*		Coeff	SE	*p*
**Average Food Security**	**−0.1141**	**0.0211**	**<0.001**	**Average Food Security**	**−0.0407**	**0.0095**	**<0.001**
CalFresh Participation	−0.1617	0.1022	0.116	CalFresh Participation	−0.0672	0.046	0.1462
**Avg Food Security × CalFresh Participation**	**0.1120**	**0.0352**	**0.0018**	**Avg Food Security × CalFresh Participation**	**0.0323**	**0.0158**	**0.0429**
**Fall 2020**				**Fall 2020—Transformed GPA**			
	**Coeff**	**SE**	***p***		**Coeff**	**SE**	***p***
**Average Food Security**	**−0.0694**	**0.0221**	**0.0021**	**Average Food Security**	**−0.0273**	**0.0097**	**0.0057**
CalFresh Participation	−0.2272	0.119	0.0586	**CalFresh Participation**	**−0.1367**	**0.0522**	**0.01**
Avg Food Security × CalFresh Participation	0.0060	0.044	0.8922	Avg Food Security × CalFresh Participation	0.0047	0.0193	0.809
